# A Modified Treatment Through Point-to-Point Coil Embolization for Direct Carotid Cavernous to Fistula: A Single-Center Result

**DOI:** 10.3389/fneur.2021.639552

**Published:** 2021-05-31

**Authors:** Zihuan Zhang, Jiaqiang Liu, Bingbing Zhang, Mengliang Zhou, Xintong Zhao, Zhenbao Li

**Affiliations:** ^1^Department of Neurosurgery, The First Affiliated Hospital of Wannan Medical College (Yijishan Hospital), Wuhu, China; ^2^Shandong Provincial Third Hospital, Cheeloo College of Medicine, Shandong University, Jinan, China; ^3^Department of Neurosurgery, Jinling Hospital, School of Medicine, Nanjing University, Nanjing, China

**Keywords:** carotid cavernous fistula, recanalization, coil embolization, fistula, neurosurgery

## Abstract

This study aims to assess the safety and efficacy of the modified treatment through point-to-point coil embolization of direct carotid cavernous fistula (dCCF), and evaluate the long-term outcome of patients who underwent the above treatment. A total of 18 patients who suffered from dCCF (a total of 19 fistulas) between January 2013 to May 2020 were analyzed. Among these patients, 14 patients were treated through point-to-point coil embolization of the fistula, while four patients were treated through combined endovascular embolization (coils, a balloon, Onyx, and/or a stent). The number of coils that filled the fistulas was counted. The primary outcome was defined by post-operative digital subtraction angiography (DSA) or the signs after the recanalization of dCCFs during the follow-up period. For patients with dCCF who underwent point-to-point coil embolization, a minimum of three coils and a maximum of 16 coils were used for these 14 fistula patients, and an average of 7.9 coils were used for each fistula, but none of the fistulas was recanalized. Furthermore, two pseudoaneurysms were observed as a result of the compression of the coils. However, none of these 14 patients presented with signs of recanalization of fistulas or cranial paralysis. The procedure applied for the present study was shown to be a safe, economical and efficacious treatment approach for dCCFs through the point-to-point coil embolization of the fistula.

## Introduction

The types of treatment for direct carotid cavernous fistula (dCCF) have been gradually developed from early internal carotid artery (ICA) occlusion and balloon embolism, to the present detachable coil combined with Onyx, covered stent treatment, flow diversion of the parent vessel, etc. However, these procedures include some drawbacks, such as recanalization, high cost and poor maneuverability. Therefore, researchers have focused on the improvement of the treatment of dCCF. The present study utilized the point-to-point coil embolization of the fistula, which is a modified form of embolization that was accidently found.

### “Point-to-Point” as a Series of Cases

#### Case 1

**Figure 1:**
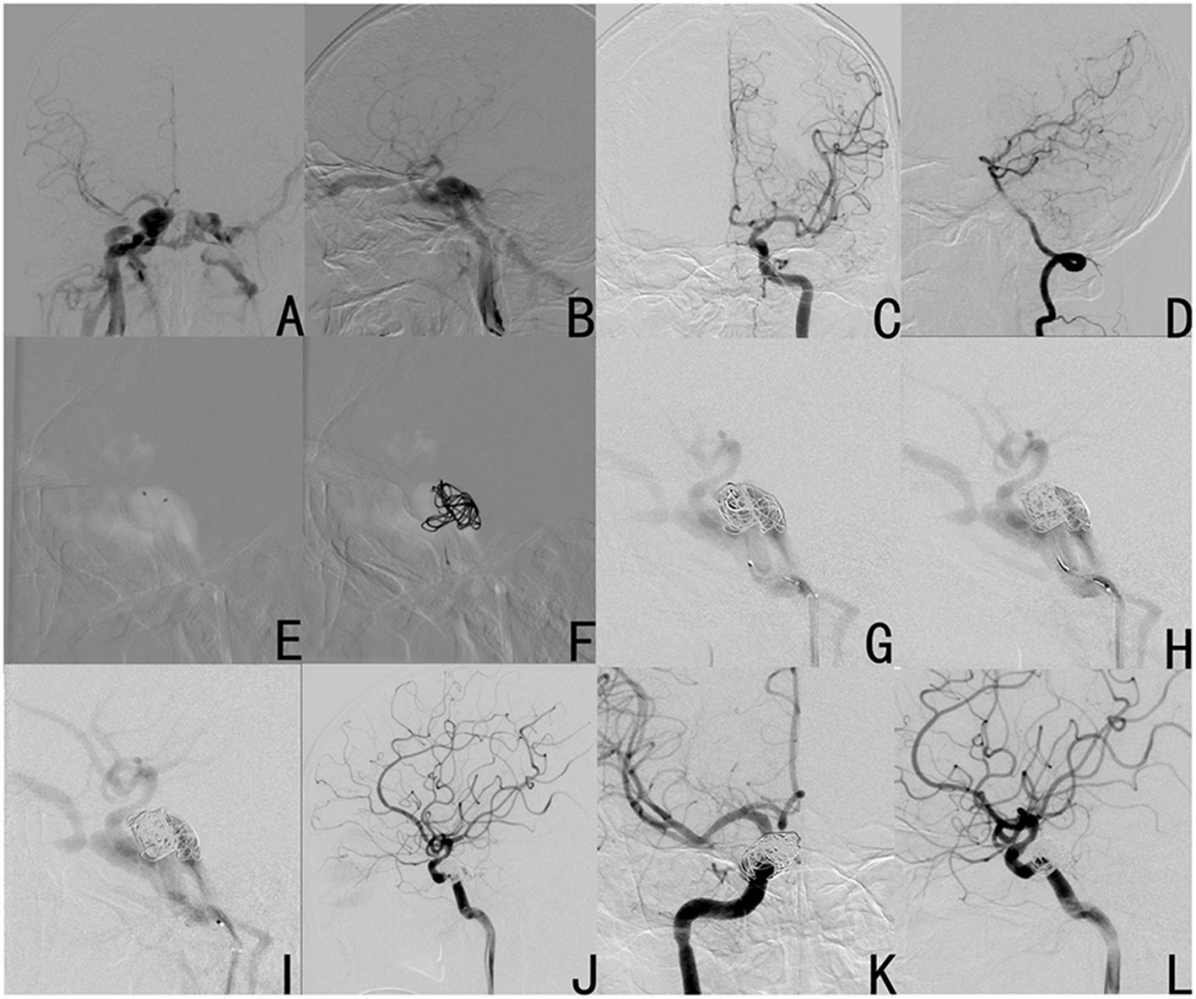
Case 1 was diagnosed with right carotid cavernous fistula (CCF) by DSA (**A**: anteroposterior view; **B**: lateral view). After temporary occlusion of the right internal carotid artery (ICA) by compression, the anterior communicating artery was not visualized after the left ICA **(C)**, and the posterior communicating artery was not visualized after the vertebral artery angiography **(D)**. Procedure: double microcatheters were put in place **(E)**; a basket with a large coil was formed **(F)**; three coils were successively filled **(G–I)**; after failure to be filled into the fistula, the 6th coil was uncoiled and withdrawn, and the fistula was completely occluded **(J)**. The fistula did not recanalize at 6 months after the operation (**K**: anteroposterior view; **L**: operation angle).

A 48-year-old male has been suffering from prominent right eye, right conjunctiva congestion, swelling and abnormal intracranial noise for 40 days after trauma. The digital subtraction angiography (DSA) revealed the presence of right carotid cavernous fistula (CCF) (Figures 1A,B). After the temporary occlusion of the right ICA by compression, the anterior communicating artery was not immediately visualized (Figure 1C), and the posterior communicating artery was also not visualized during the vertebral artery angiography (Figure 1D). In addition, the patient could not tolerate the temporary right internal carotid compression test (<2 min) and suffered syncope. If Onyx was used for embolization, a filling balloon needs to be used to temporally occlude the fistula, which would prevent the Onyx from filtrating into the internal artery. However, the filling balloon would block the blood flow of the internal artery, and the blocking time would last for more than 2 min, which may lead to cerebral ischemia. Therefore, Onyx embolization was not suitable for this patient due to lack of good compensation. Simple coil embolization was chosen, because the patient could not afford the high cost. Larger coils were preferred to reduce the number of coil usage. During the operation, double microcatheters were placed, and the first five coils were smoothly filled (Figures 1E–I). When the 6th coil was filled, the fistula could not be completely filled, although the attempt to adjust the coil was made. Hence, the 6th coil was withdrawn. Due to the withdrawal of the 6th coil, the originally filled five coils were filled closely toward the fistula. The right ICA showed complete occlusion of the fistula and patency of the parent artery (Figure 1J). After 6 months, the DSA revealed no recanalization of the fistula (Figures 1K,L). After 7 years of follow-up, no symptoms of recanalization occurred. Therefore, the good outcome of this patient suggests that this procedure is feasible for treating the dCCF by simple coil embolization of the fistula.

## Materials and Methods

### Demographics and Clinical Characteristics of Patients

The present study was carried out in accordance to the Declaration of Helsinki guidelines, and was approved by the Ethics Committee of The First Affiliated Hospital of Wannan Medical College (Yijishan Hospital). The duration of the study was carried out for a period of 8 years, between January 2013 and May 2020. For the present study, a total of 18 patients with 19 dCCFs were recruited. Among those patients, 14 patients (seven males and seven females) with 15 dCCFs were successfully treated through point-to-point coil embolization of the fistula. The remaining four patients (two males and two females) with four dCCFs were treated with the combined endovascular embolization (coils, a balloon, Onyx, and/or a stent), and these served as the controls. The balloon used for the Onyx embolization was a non-detachable balloon (HyperForm Occlusion Balloon). The stent used for the present study was a LVIS stent. All patients had symptoms, and all 19 dCCFs were placed under the type A category. Type A fistulas have direct communication between the ICA and cavernous sinus, and are usually associated with high flow rates (1). Direct type A CCFs are considered to occur secondary to traumatic injury, iatrogenic injury, or a form of spontaneous fistula. The most common type is traumatic CCF, and this usually occurs due to a motor vehicle accident, with a closed traumatic brain injury that involves a basilar skull fracture. Most spontaneous CCFs occur due to post-rupture of a pre-existing intracavernous carotid aneurysm, and this may also be associated with the intrinsic weakness of the vessel wall caused by some genetic diseases, to a lesser extent, such as Ehlers-Danlos type IV and fibromuscular dysplasia (2). An equal number of males (*n* = 9) and females (*n* = 9) were recruited, and the mean age was 51.83 ± 3.00 years old (age range: 21–79 years old). The ratio of the right to the left CCF was 11:8. The main associated signs and symptoms were headaches, exophthalmos, bulbar conjunctival hyperemia, and abnormal vascular bruit. The clinical characteristics in the point-to-point coil embolization group and control group are presented in Tables 1, 2, respectively.

**Table 1:** Summary of patients with direct CCFs who underwent point-to-point coil embolization of the fistula.

**Case**	**Gender**	**Age**	**Diagnosis**	**Symptomatic (Y/N)**	**Embolization time**	**Number of coils**	**Outcomes**
1	M	48	Right CCF	Y	2013.01	5	Excellent
2	F	62	Left CCF	Y	2013.09	6	Excellent
3	M	46	Right CCF	Y	2014.01	10	Excellent
4	F	62	Left CCF	Y	2014.08	16	Excellent
5	M	79	Right CCF	Y	2014.11	6	Excellent
6	M	36	Left CCF	Y	2015.01	4	Excellent*
7	M	51	Right CCF	Y	2015.04	10	Excellent
8	F	45	Bilateral CCF	Y	2015.10 (R)	10	Excellent
					2015.11 (L)	8	Excellent
9	F	61	Left CCF	Y	2016.02	9	Excellent
10	M	55	Right CCF	Y	2017.08	3	Excellent*
11	F	50	Left CCF	Y	2017.09	8	Excellent
12	F	35	Right CCF	Y	2018.06	9	Excellent
13	M	24	Right CCF	Y	2020.01	8	Excellent
14	F	63	Left CCF	Y	2020.02	3	Excellent

Case 8 with bilateral CCFs was treated on October 2015 (right CCF) and November 2015 (left CCF). For case 6 and 10, the post-operative DSA revealed the complete occlusion of the fistulas, except for the compression of the coils (indicated by *).

**Table 2:** Summary of patients with direct CCFs, who underwent combined endovascular embolization.

**Case**	**Gender**	**Age**	**Diagnosis**	**Symptomatic (Y/N)**	**Embolization time**	**Embolization materials**	**Outcomes**
15	M	47	Right CCF	Y	2014.01	Coil*4+balloon*1+onyx*1+stent*1	Excellent
16	F	51	Right CCF	Y	2019.01	Coil*9+balloon*1+onyx*2+stent*1	Excellent
17	M	54	Left CCF	Y	2020.05	Coil*17+balloon*1+onyx*1	Excellent
18	F	64	Right CCF	Y	2019.07	Coil*8+balloon*1+onyx*1	Recanalization
Secondary operation				Y	2019.09	Coil*3+balloon*1+onyx*1	Excellent

### Imaging Examination

DSA was pre-operatively performed for all 18 patients who suffered from CCFs, and one of these patients presented with bilateral CCFs.

### Treatment Strategy: “Point-to-Point” as a Series of Cases

**Figure 2:**
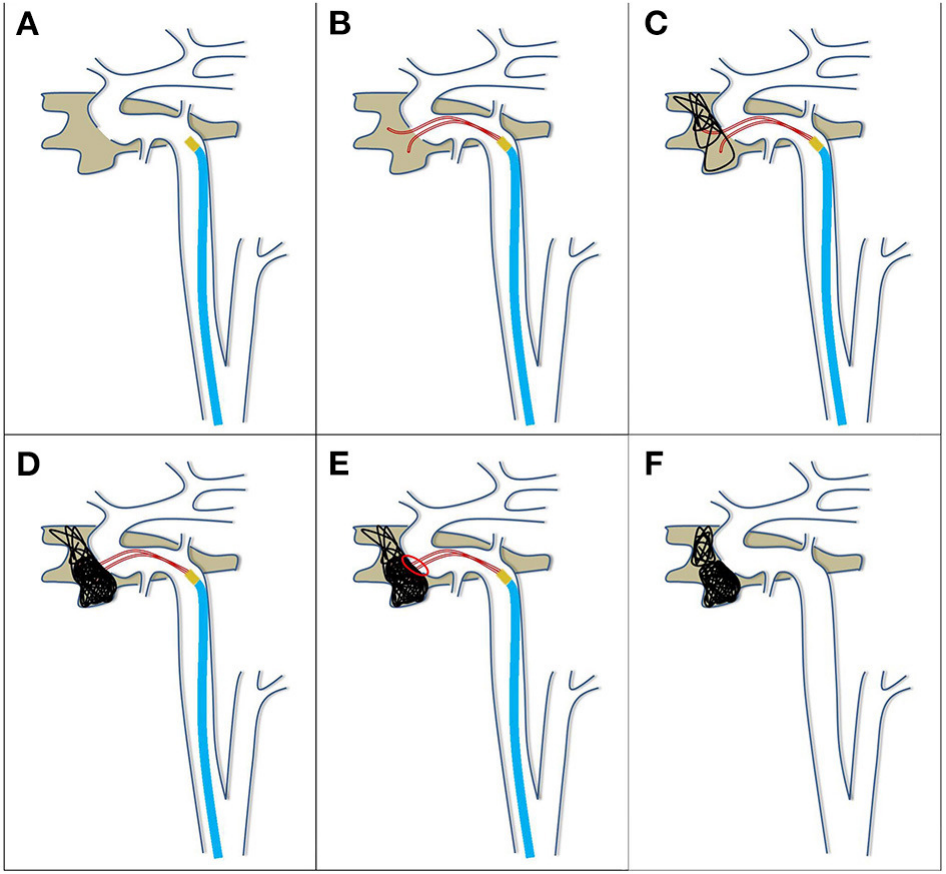
Schematic diagram of the treatment for direct CCF by point-to-point coil embolization of the fistula. Procedure: first, the location of the fistula was accurately determined **(A)**; second, the double microcatheters were preset, one shallow and one deep **(B)**; third, a basket with a large coil was formed by the deep microcatheter **(C)**; fourth, the coils were filled into only the part close to the fistula, until tight filling by the shallow microcatheter **(D)**; fifth, the microcatheters were withdrawn, and the closure of the fistula was confirmed **(E,F)**.

The specific procedures for the “point-to-point” coil embolization is described in Figure 2. In order to carry out the procedure, the first initial step was to accurately determine the location of the fistula (which was called the one “point,” Figure 2A). Then, the double microcatheter technique was used, in which one microcatheter was placed in the shallow position, while another microcatheter was placed in the deep position (second step, Figure 2B). Afterwards, the large coil was used to form a basket to support the deep microcatheter (third step, Figure 2C). Subsequently, coils were used to only fill parts that were close to the fistula, until tight filling was attained by the shallow microcatheter (which was called another “point,” Figure 2D). Finally, the closure of the fistula was confirmed. Even if the fistula still presented and developed, the blood flow rate remained very low, the retention of the contrast agent was obvious, and the thrombosis led to complete occlusion of the fistula after 5–10 min. Sometimes, the fistula would be recanalized after the withdrawal of the microcatheter. Thus, it is essential to observe this for another 5–10 min (Figures 2E,F).

The combined endovascular embolization was performed for the control cases. In addition to the use of coils, ethylene vinyl alcohol copolymer (Onyx) was used through a HyperForm Occlusion Balloon (EV3, USA). For case 15 and 16, the Onyx was infiltrated in the internal carotid artery, and a LVIS stent (MicroVention, USA) was used to compress and fix the Onyx to the vessel wall, respectively.

### Statistical Analysis

The statistical data was analyzed using SPSS version 23 (IBM, USA). The numerical data was presented as mean ± standard deviation (SD), and student's *t*-test was used to compare the statistical analysis of the two groups. The categorical data was summarized, and presented in number and percentage. Comparisons between the two groups were performed using Chi-square-test for categorical data. *P* < 0.05 was considered statistically significant.

## Results

For patients with dCCF, who underwent point-to-point coil embolization, the fistulas were successfully occluded with simple coil embolization, without stent or balloon assistance. Post-operative DSA was performed, and it was found that the fistulas were embolized. At the same time, the above symptoms and signs were relieved. During the mean follow-up of 51.6 months, two patients presented with coil compression (case 6 and 10), while none of the other patients presented with recanalization of the fistula or any cranial paralysis (Table 1). Among the 14 patients who underwent point-to-point coil embolization, 11 patients were confirmed to have no recanalization of the fistula by angiography, and three patients reported and confirmed that they had no related symptoms by telephone follow-up. If recanalization occurred, symptoms and signs would recur. Three patients could not return to the hospital for follow-up due to reasons, such as low income, lack of willingness, and lack of knowledge of the disease. Although these patients did not undergo DSA, the symptoms and signs did not recur. Therefore, it could be determined that recannalization did not occur. For the four patients with dCCF who underwent combined endovascular embolization, one patient developed recanalization and underwent a secondary operation after 2 months (Table 2), and the remaining three patients had no recanalization, which was confirmed by angiography.

A summary of all 18 patients with dCCF is presented in Table 3. For the 14 patients who under point-to-point coil embolization, seven (50%) patients were male and seven (50%) patients were female. For the control patients, two (50%) patients were male and two (50%) patients were female. Patients in the point-to-point coil embolization group had a mean age of 51.21 years old, while patients in the control group had a mean age of 54.00 years old. In the point-to-point coil embolization group, seven (46.67%) patients had CCF on the left side, and eight (53.33%) patients had CCF on the right side. In the control group, one (25.00%) patient had CCF on the left side and three (75.00%) patients had CCF on the right side. Recanalization was a treatment failure in the present study, and one (25%) patient from the control group presented this. None of the patients who underwent point-to-point coil embolization developed recanalization (0%). The point-to-point coil embolization lead to a reduced rate of treatment failure, when compared to the control group (*P* < 0.05).

**Table 3:** Clinical characteristics and outcomes of patients with direct CCFs, who underwent point-to-point coil embolization of the fistula or the combined endovascular embolization.

**Variables**	**Classification**	**Embolization methods**		***X*^**2**^/*t***	***P***
		**Point-to-point coil embolization**	**Controls**		
Gender (%)	Male	7 (50.00)	2 (50.00)	0	>0.999
	Female	7 (50.00)	2 (50.00)		
Age (mean ± SD)		51.21 ± 14.05	54.00 ± 7.26	0.377	0.712
CCF side (%)	Left	7 (46.67)	1 (25.00)	0.608	0.436
	Right	8 (53.33)	3 (75.00)		
Treatment failure (%)	No	14 (100)	3 (75.00)	3.706	<0.05
	Ye	0 (0)	1 (25.00)		

### Illustrative Cases: “Point-to-Point” as a Series of Cases

#### Case 7

**Figure 3:**
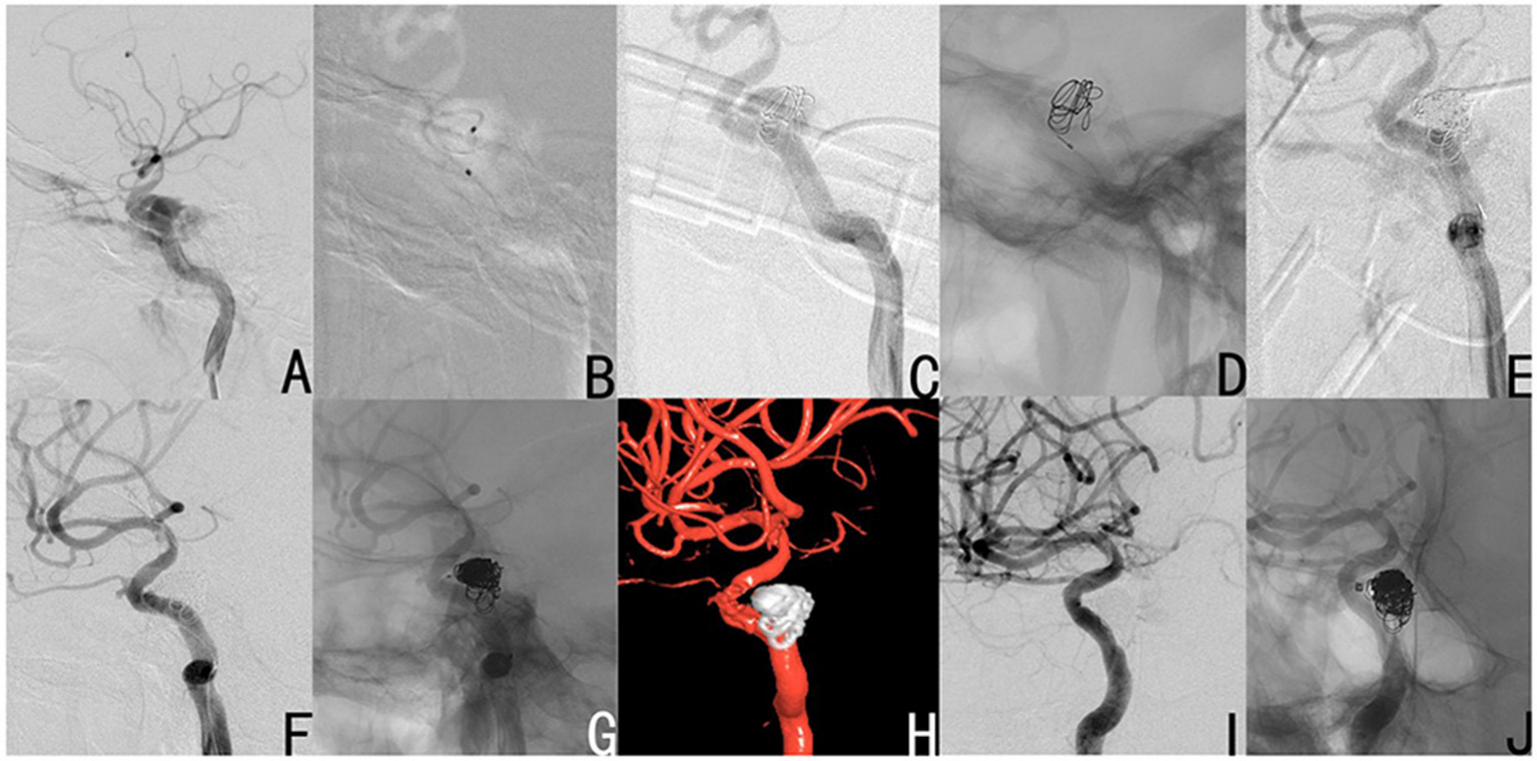
Case 7 had a right CCF (**A**: lateral view). After the location of the fistula was determined, the deep microcatheter was put in place **(B)**, and a large coil was used to form a basket as a support **(C,D)**. Then, the other coils were filled into the shallow part of the fistula by the shallow microcatheter **(E)**, until the fistula disappeared **(F)**. The angiography without subtraction **(G)** and 5s-3D angiography **(H)** revealed that these coils were stable and tightly covered the fistula, dense in the shallowness of the fistula, and loose in the depth of the fistula. At 18 months after the operation, the DSA revealed no recanalization of the fistula, excellent reconstruction of the parent vessel **(I)**, and the stable shape of these coils **(J)**.

A 51-year-old male subject was referred to our institution after the onset of frequent headaches, abnormal vascular bruit, and the absence of vision in the right eye. The preoperative DSA revealed the existence of right CCF (Figure 3A). However, after identifying the location of the fistula, one microcatheter was preset into the depth of the fistula (Figure 3B). Then, a large coil was used to form a basket, and support the microcatheter (Figures 3C,D). Accordingly, another microcatheter was placed in the shallow part of the fistula, and the coils were filled into this region through the microcatheter (Figure 3E), until the fistula was occluded (Figure 3F). The angiography without subtraction (Figure 3G) and 5s-3D angiography (Figure 3H) revealed that these coils remained stable, and tightly covered the fistula. Furthermore, these were dense in the shallowness of the fistula, and loose in the depth of the fistula. At 18 months after the operation, the DSA revealed no recanalization of the fistula, the excellent reconstruction of the parent vessel (Figure 3I), and the stable shape of the coils (Figure 3J).

#### Case 8

**Figure 4:**
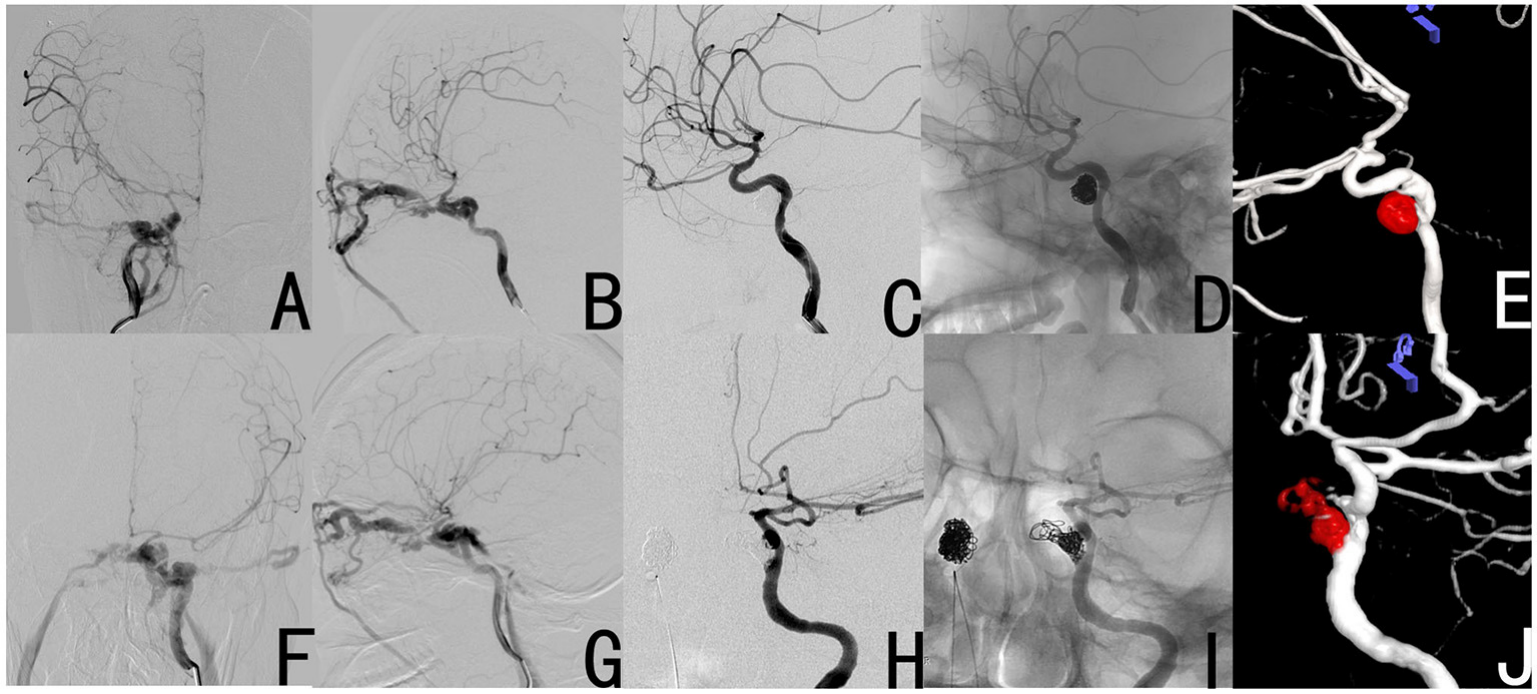
Case 8 had bilateral CCFs, and these were completely occluded by simple coil embolization of the fistula. The preoperative DSA revealed the right CCF **(A,B)**. The post-operative DSA revealed that the fistula disappeared **(C–E)**. The preoperative DSA revealed the left CCF **(F,G)**. The post-operative DSA revealed that the fistula disappeared **(H–J)**.

A 45-year female subject became unconscious after a traffic accident. The patient subsequently presented with bilateral exophthalmos, bulbar conjunctival hyperemia, and abnormal vascular bruit. The DSA exhibited bilateral CCFs (right CCF: Figures 4A,B; left CCF: Figures 4F,G). These bilateral CCFs were completely occluded through point-to-point coil embolization of the fistula on October 2015 (right CCF: Figures 4C–E) and November 2015 (left CCF: Figures 4H,J). During the follow-up period, the subject did not present with any symptoms of recanalization of the fistula.

#### Case 10

**Figure 5:**
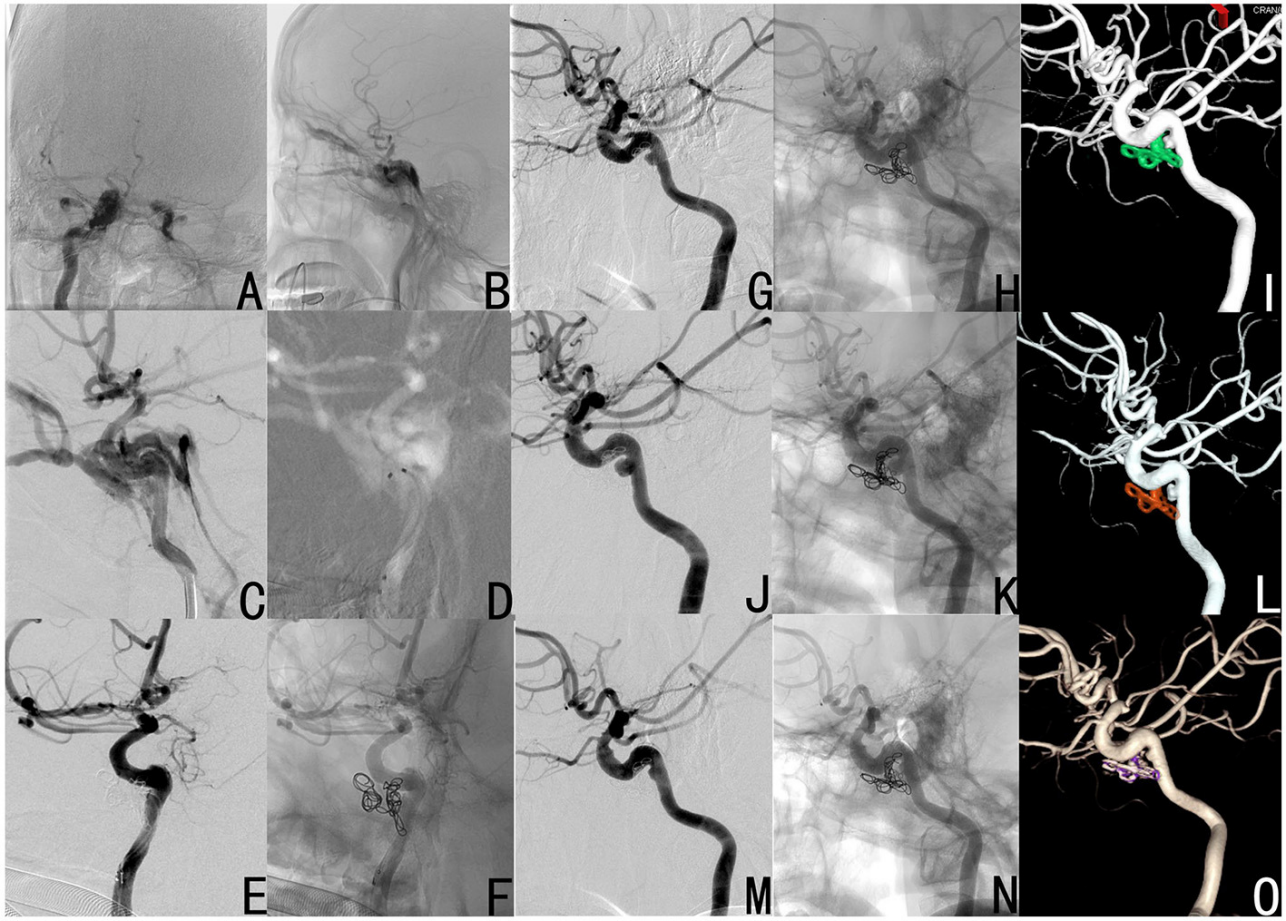
Case 10 was diagnosed with right CCF by DSA (**A**: anteroposterior view; **B**: lateral view), and was treated by simple coil embolization by only using three coils (**C**: operation angle for the location of the fistula; **D**: double microcatheters were put in place; **E,F**: the occlusion of the fistula). At 3 months **(G–I)**, 1 year **(J–L)** and 2 years **(M–O)** after the operation, the DSA revealed a pseudoaneurysm, which was the result of the compression of coils, but the fistula was always not recanalized. Furthermore, the pseudoaneurysm initially became enlarged, and subsequently dwindled.

A 55-year-old male subject presented with abnormal vascular bruit after trauma. The DSA revealed a right CCF (Figures 5A,B), and this was successfully occluded through the above treatment using only three coils (Figures 5C–F). At 3 months after the operation, the DSA revealed a pseudoaneurysm as a result of the compression of coils, but there was no recanalization of the fistula (Figures 5G–I). At 1 year after the operation, the DSA revealed the enlargement of the pseudoaneurysm as a result of the further compression of coils. However, no recanalization of the fistula was observed (Figures 5J–L). At 2 years after the operation, the DSA showed that the pseudoaneurysm became much smaller than the earlier size, and the fistula was still occluded (Figures 5M–O).

## Discussion

At present, there are different kinds of advanced treatments available for treating CCFs, such as balloon embolization (3), covered stents (4), and detachable coils combined with Onyx embolization (5). However, none of these were found to be reliable. Balloon embolization has been used in practice due to its advantages, such as practicality and keeping the ICA unobstructed. However, at present, this is seldom used due to its high recanalization of fistulas (the balloons are punctured and leaks) and poor controllability (accidental falling off of the balloon). Covered stents are relatively hard, and may induce vasospasm (1), especially for patients suffering from Ehlers Danlos syndrome. Therefore, there is an increased risk of ICA dissection and rupture (6–8). Due to its high maneuverability and definite efficacy, detachable coil combined with Onyx embolization has been widely used. However, there are two drawbacks for this treatment: one drawback is the temporary block of blood flow due to balloon filling during the operation, which is sometimes not tolerated by some patients (for example, Case 1), and the other one is that the Onyx might inevitably flow back to the ICA, and may cause cerebral infarction. In addition, the onset of cranial nerve (CN III, CN V, CN VI, and/or CN VII) palsy has been reported after the occurrence of transvenous embolization or transarterial embolization of CCFs with Onyx (9).

It has been understood that simple coil embolization can be used to treat CCFs. However, a large number of coils are needed to fill the fistula (10). In addition, the coil embolization of CCFs has been reported to cause cranial neuropathies (10), which is more frequent than liquid embolism, due to the mass effect and/or thrombogenic effect (1). In another study, Kashiwazaki et al. asserted the greater overall coil length and density of the coiling of the posterior portion of the cavernous sinus in patients who developed CN VI paresis (11). However, Bou Ghannam et al. reported that CN III and CN VI paresis presented at 4–5 years after the endovascular coiling of CCFs (12).

Although the point-to-point coil embolization of dCCF applied in the present study contained no balloons, covered stents, or liquid embolism, the number of coils used was very small (an average of 7.7 per fistula), and merely three coils were used to the minimum. Furthermore, none of the patients suffered from cranial paresis symptoms. This can be explained by the low usage of coils, which lead to a low mass effect. In addition, these coils are affordable. Therefore, the cost and duration of hospitalization might be reduced, when compared with the combined treatment, due to the lower usage of coils.

### Limitations of the Point-to-Point Coil Embolization

First, the point-to-point coil embolization of the fistula is not suitable for multiple fistulas, when present on the same side or dural CCF. Second, the sample size of the present study was small, which may contribute to an insignificant difference in the incidence of recanalization after embolization using different methods. In addition, no differences in treatment effect by gender were found during the analysis.

### Analysis of the Treatment Failure

After providing treatment through point-to-point coil embolization, the DSA exhibited two pseudoaneurysms, which resulted from the compression of coils in case 6 and 10. The number of filled coils was four for case 6 and three for case 10. This could be due to the low density of coiling, which was the result of the small number of coils that compressed the coils, and the occurrence of pseudoaneurysms. Interestingly, it was observed that one of the two pseudoaneurysms initially increased, and subsequently decreased (case 10). However, to the satisfaction of the investigators, all 15 fistulas in the 14 patients were not recanalized during the follow-up period.

During the treatment with combined embolization for case 15 and 16, the Onyx leaked into the internal carotid artery. Therefore, a LVIS stent was used to stabilize the Onyx cast within the internal carotid artery, preventing the Onyx cast from occluding the vessel and causing cerebral ischemia. This was not considered as a complication, since this occurred during, but not after, the embolization. After treatment with the combined embolization, the DSA showed that case 18 developed recanalization. This patient underwent secondary embolization at 2 months after the initial embolization, and developed no further recanalization.

It can be concluded that point coil embolization is an safe treatment procedure, economical, and a efficacious treatment for dCCF through “point-to-point,” as a series of cases to treat coil embolization of the fistula. Without doubt, a randomized clinical trial with a larger sample size, and a comparative efficacy and cost analysis needs to be performed. In future randomized controlled clinical trials, the investigators will make the inclusion criteria stricter, and include patients suitable for both point-to-point embolization and combined embolization.

## Data Availability Statement

The raw data supporting the conclusions of this article will be made available by the authors, without undue reservation.

## Ethics Statement

The studies involving human participants were reviewed and approved by The Present Study was carried out in accordance with the Declaration of Helsinki guidelines, and was approved by the Ethics Committee of Yijishan Hospital of Wannan Medical College. The patients/participants provided their written informed consent to participate in this study. Written informed consent was obtained from the individual(s) for the publication of any potentially identifiable images or data included in this article.

## Author Contributions

ZZ performed the studies and wrote the manuscript. JL and BZ participated in drawing the images. MZ performed the data analysis. XZ and ZL contributed to the design and analysis of the study. All authors contributed to the article and approved the submitted version.

## Conflict of Interest

The authors declare that the research was conducted in the absence of any commercial or financial relationships that could be construed as a potential conflict of interest.
